# Nationwide Surveillance of Clinical Carbapenem-resistant Enterobacteriaceae (CRE) Strains in China

**DOI:** 10.1016/j.ebiom.2017.04.032

**Published:** 2017-04-26

**Authors:** Rong Zhang, Lizhang Liu, Hongwei Zhou, Edward Waichi Chan, Jiaping Li, Ying Fang, Yi Li, Kang Liao, Sheng Chen

**Affiliations:** a Second Affiliated Hospital of Zhejiang University, Hangzhou, PR China; b Shenzhen Key Lab for Food Biological Safety Control, Food Safety and Technology Research Center, Hong Kong PolyU Shen Zhen Research Institute, Shenzhen, PR China; c State Key Lab of Chirosciences, Department of Applied Biology and Chemical Technology, The Hong Kong Polytechnic University, Hung Hom, Kowloon, Hong Kong; d Clinical Laboratory, Medicine Department, He Nan Provincial People's Hospital, Zhengzhou, PR China; e First Affiliated Hospital, Sun Yat-Sen University, Guangzhou, PR China

**Keywords:** Carbapenem resistance, Enterobacteriaceae, Plasmid, *bla*_NDM_, *bla*_KPC-2_, Molecular epidemiology

## Abstract

The increasing incidence of carbapenem-resistant Enterobacteriaceae (CRE) - mediated hospital infections in China prompted a need to investigate the genetic basis of emergence of such strains. A nationwide survey was conducted in China covering a total of 1105 CRE strains collected from 25 geographical locales with results showing that acquisition of two carbapenemase genes, *bla*_KPC-2_ and *bla*_NDM_, was responsible for phenotypic resistance in 90% of the CRE strains tested (58% and 32% respectively), among which several major strain types, such as ST11 of *K. pneumoniae* and ST131/ST167 of *E. coli*, were identified, suggesting that dissemination of specific resistant clones is mainly responsible for emergence of new CRE strains. Prevalence of the *fosA3* gene which mediates fosfomycin resistance, was high, while the colistin resistance determinant *mcr-1* was rarely present in these isolates. Consistently, the majority of the *bla*_NDM_-bearing plasmids recoverable from the test strains belonged to IncX3, which contained a common core structure, *bla*_NDM_-*blaMBL*-*trpF*. Likewise, the core structure of IS*Kpn27*-*bla*_KPC-2_-IS*Kpn2* was observed among plasmids harboring the *bla*_KPC-2_ gene, although they were genetically more divergent. In conclusion, the increasing prevalence of CRE strains in China is attributed to dissemination of conservative mobile elements carrying *bla*_NDM_ or *bla*_KPC-2_ on conjugative and non-conjugative plasmids.

## Highlights

• Over 90% of CRE strains in China harbored the *bla*_NDM_ or *bla*_KPC-2_ gene.
• The predominant types of carbapenem-resistant *K. pneumoniae* and *E. coli* were ST11 and ST131/ST167 respectively.
• Conservative mobile elements carrying *bla*_NDM_ and *bla*_KPC-2_ contributed to transmission of carbapenemase genes in CRE.

Large scale surveillance of CRE was rarely reported worldwide except for a recent survey reported in European countries. A nationwide survey was conducted in this study to depict the epidemiological features of CRE strains in China and the types of resistance elements that they harbored. We showed that the increasing prevalence of CRE strains in China was due to dissemination of major resistant clones that harbor several major types of *bla*_NDM_ or *bla*_KPC-2_ – bearing conjugative plasmids, and that infections caused by such strains remain treatable by various non-β-lactam antibiotics, including colistin.

## 1 Introduction

The scale of clinical and public health problems due to multidrug-resistant bacterial infections has further escalated in recent years following the emergence of *bla*_NDM_, a plasmid-borne carbapenem resistance gene that has been widely disseminated among various species of bacterial pathogens worldwide (Kumarasamy et al., 2010, Nordmann et al., 2012). Descriptions such as “superbug”, “nightmare bacteria” and “post-antibiotic era” reflected the seriousness of the antimicrobial resistance issue.

Among the major multidrug-resistant organisms that emerged within the past two decades, carbapenem-resistant *Enterobacteriaceae* (CRE), which commonly cause untreatable and hard-to-treat infections among hospitalized patients, is considered an urgent threat according to a report by the Center for Diseases Control and Prevention (CDC) in 2013 on antibiotic resistance threats in the United States. In the past two decades, utilization of carbapenems such as imipenem and meropenem in clinical treatments has become necessary due to proliferation of multidrug-resistant bacterial pathogens in clinical settings (Zilberberg and Shorr, 2013, Goel et al., 2011). Such increase in carbapenem consumption has been accompanied by the emergence of carbapenem-resistant Gram-negative pathogens (Karaiskos and Giamarellou, 2014, Livermore, 2004, Livermore, 2009). According to the CDC report of 2013, > 9000 healthcare-associated infections are caused by CRE each year and almost half of the hospital patients who suffer from CRE-mediated bloodstream infections died subsequently (CDC, 2013). Each year, approximately 600 deaths result from infections caused by the two most common types of CRE, namely carbapenem-resistant *Klebsiella* spp. and *E. coli* (Yong et al., 2009).

In China, the first clinical report of *bla*_NDM_ involved carbapenem-resistant *Acinetobacter baumannii* strains detectable in four patients who resided in different provinces, in 2011 (Chen et al., 2011). Since then it has been recoverable in most species of *Enterobacteriaceae*, including *K. pneumoniae*, *Klebsiella oxytoca*, *Escherichia coli*, *Enterobacter cloacae*, *Enterobacter aerogenes* and *Citrobacter freundii*, in various cities or regions in China, such as Beijing, Changsha, Chongqing, Fuzhou, Guangzhou, Hangzhou, Hebei, Hong Kong and Zhengzhou (Berrazeg et al., 2014, Qin et al., 2014). The first KPC producing CRE strain in China was reported in 2007, and the *bla*_KPC-2_ gene has since become the most widely spread carbapenemase gene in China as well as various parts of the world. In this study, we conducted a nationwide surveillance of the prevalence of CRE in China and investigated the molecular epidemiological features of these strains, and hoped to identify the key strains and mobile resistance elements responsible for causing an increase in prevalence of CRE-mediated infections in China. Findings of this work shall provide essential insight into development of effective strategies for worldwide control of CRE and reducing the rate of untreatable infections in clinical settings.

## 2 Materials and Methods

### 2.1 Carbapenem-resistant Enterobacteriaceae Isolates

Non-duplicated *Enterobacteriaceae* strains that exhibited carbapenem resistance phenotype (meropenem MIC ≥ 4 μg/ml) were collected from hospitals located in 25 Provinces and Municipalities in China, namely Anhui, Beijing, Fujian, Gansu, Guangdong, Guangxi, Guizhou, Hainan, Hebei, Henan, Hubei, Hunan, Jilin, Jiangxi, Liaoning, Nanjing, Shandong, Shanxi, Shaanxi, Shanghai, Sichuan, Tianjing, Xinjiang, Zhejiang and Chengdu, during the period, June 2014 through June 2015. One representative hospital (normally the largest general hospital in the location) from each location was chosen for sample collection. All strains were subjected to species confirmation using the Vitek 2 system (bioMérieux, Marcy-l'E' toile, France), and the MALDI-TOF MS apparatus (Bruker Microflex LT, Bruker Daltonik GmbH, Bremen, Germany).

### 2.2 Antimicrobial Susceptibility Testing

The minimal inhibitory concentrations (MICs) of 12 antibiotics, namely amoxicillin-clavulanic acid, cefotaxime, ceftazidime, imipenem, mropenem, amikacin, ciprofloxacin, colistin, fosfomycin and tigecycline, were determined using the agar dilution method, and the results were analyzed according to the CLSI criteria of 2016 (Huang et al., 2016, CLSI, 2016). The 2017 EUCAST breakpoints were used (available at http://www.eucast.org/clinical_breakpoints/) for tigecycline.

### 2.3 Screening of Carbapenemase and Other Antimicrobial Resistance Genes

PCR and nucleotide sequencing were performed to screen for the presence of the carbapenemase-encoding genes *bla*_VIM_, *bla*_IMP_, *bla*_KPC_, *bla*_OXA-48_ and *bla*_NDM_ as described previously (Dallenne et al., 2010). Screening of *fosA3* and *mcr-1* was performed as previously described (Li et al., 2016, Li et al., 2017, Liu et al., 2017, Lin and Chen, 2015). An imipenem-EDTA double-disc synergy test and the modified Hodge test were used to assess the ability of the test strains to produce carbapenemases; analysis was performed according to CLSI guidelines (Huang et al., 2016, CLSI, 2016).

### 2.4 PFGE and ST Typing

Multi locus sequence typing (MLST) for these CRE isolates was performed according to the previously reported protocol (Liu et al., 2014). Clonal relationships of major ST stain types of *K. pneumonia* and *E. coli* were investigated by PFGE of *Xba*I-digested genomic DNA using a Rotaphor System 6.0 instrument (Whatman Biometra, Goettingen, Germany), with a running time of 24 h and pulse times of 3–40 s. *Salmonella* strain H9812 was used as the control strain. Dendrograms depicting the genetic relatedness of the test strains were generated from the homology matrix to describe the relationships of the PFGE profiles of the test strains.

### 2.5 Conjugation, S1-PFGE and Southern Hybridization

Conjugation experiments were carried out using the mixed broth method as previously described (Borgia et al., 2012). PFGE, S1-PFGE and Southern Hybridization were performed as previously described (Wang et al., 2015).

### 2.6 Plasmid Sequencing

Plasmids carrying the *bla*_KPC-2_ and *bla*_NDM_ genes were extracted from transformants using the Plasmid Midi kits (Qiagen, Germany). The plasmids were subjected to sequencing using Illumina NextSeq 500 platforms. After obtaining the raw reads, SPAdes was utilized to perform the hybrid-assembly and obtain complete plasmid sequences. Illumina short-reads were then utilized to polish the finished plasmids. The RAST annotation pipeline was chosen to perform rapid annotation of the plasmids (Overbeek et al., 2014). Comparison of the plasmids against the highly homologous plasmids in the NCBI database was performed by BRIG (Alikhan et al., 2011).

### 2.7 Plasmid Mapping

PCR mapping of the conservative regions of IncX3 plasmid and regions carrying *bla*_NDM_-bearing mobile elements was performed on IncX3 plasmids as previously described (Huang et al., 2016). The genetic environment of *bla*_KPC-2_ on conjugative plasmids was analyzed by primer walking as previously described (Pfeifer et al., 2011).

## 3 Results

### 3.1 CRE Strains and Their Susceptibility to Various Antimicrobials

A total of 1105 non-duplicate CRE strains collected from hospitals in 25 Provinces and Municipalities in China were studied to obtain molecular epidemiological features of such organisms. *K. pneumoniae* was the most prevalent species (703 strains), followed by *E. coli* (164), *E. cloacae* (132), *E. aerogenes* (Alikhan et al., 2011), *Klebsiella oxytoca* (Alikhan et al., 2011), *Serratia marcescens* (Borgia et al., 2012), *C. freundii* (Borgia et al., 2012) and 16 strains of other *Enterobacteriaceae* species (Table 1). All carbapenem-resistant *K. pneumoniae*, *E. coli* and *E. cloacae* isolates were found to be resistant to almost all β-lactam antibiotics tested, with only a small proportion of the strains being susceptible to carbapenems and cephalosporins. The rate of susceptibility to amikacin, ciprofloxacin, fosfomycin and tigecycline were respectively 47.7%, 27.7%, 31.3% and 7.8% among the *K. pneumoniae* strains, 68.8%, 41.4%, 88.9% and 54.4% among the *E. coli* strains, and 62.5%, 25.0%, 35.3% and 6.8% among the *E. cloaceae* strains. Overall, resistance to colistin was extremely rare among CRE strains in China, with respectively 1.1%, 2.3% and 6.2% of the *K. pneumoniae*, *E. coli* and *E. cloaceae* strains displaying colistin MIC ≧ 4 μg/ml (Table 2).

**Table 1:** Prevalence of different carbapenemse genes harbored by 1105 clinical CRE strains recovered from various geographical locations in China.

Bacterial species	Total no.	No. of *bla*_KPC_ positive strains	No. of *bla*_NDM_ positive strains	No. of *bla*_KPC_ + *bla*_NDM_ positive strains	No. of *bla*_IMP_ positive strains	No. of strains carrying carbapenemase genes
*K. pneumoniae*	703	517 (74%)	121 (17%)	10	19	668 (95%)
*E. coli*	164	65 (40%)	81 (49%)		3	150 (91%)
*E. cloacae*	132	19 (14%)	81 (61%)	1	4	105 (80%)
*K. oxytoca*	24	7 (29%)	10 (42%)	1	6	24 (100%)
*E. aerogenes*	24	4 (17%)	16 (67%)	4		24(100%)
*S. marcescens*	21	6 (29%)	14 (67%)	1		21(100%)
*C. freumdii*	21	2 (9%)	13 (62%)	4	2	21(100%)
Others^a^	16	7 (44%)	7 (44%)	0	0	12 (88%)
Total	1105	627 (57%)	343 (31%)	21	35	887 (93%)

a Other Enterobacteriaceae included *M. morganii*, *P. mirabilis*, *R. ornithinolytica*, and *L. adecarboxylata.*

**Table 2:** Antimicrobial susceptibility profiles of clinical carbapenem-resistant *K. pneumoniae*, *E. coli* and *E. cloacae* strains.

Antibiotics	*K. pneumoniae*			*E. coli*			*E. cloacae*		
	Rate (%)			Rate (%)			Rate (%)		
	S	I	R	S	I	R	S	I	R
Amoxicillin-clavulanic acid	9.4	0.03	90.2	2.3	0	97.7	1.0	0	99.0
Cefotaxime	0	0	100	0	0	100	0	0	100.0
Ceftazidime	1.0	1.4	97.6	1.6	0.8	97.7	1.0	4.2	94.8
Imipenem	2.7	2.4	94.9	3.1	5.5	91.4	2.1	6.3	91.7
Meropenem	2.9	1.9	95.3	3.9	2.3	93.8	2.1	3.1	94.8
Amikacin	47.7	0.7	51.6	68.8	0	31.3	62.5	4.2	33.3
Ciprofloxacin	27.7	5.6	66.8	41.4	1.6	57.0	25.0	8.3	66.7
Colistin	96.1	2.8	1.1	97.7	0	2.3	93.8	0	6.2
Fosfomycin	31.3	6.8	61.9	88.9	0	11.1	35.3	3.5	61.2
Tigecycline	7.8	68.5	23.7	54.4	39.45	6.1	6.8	36.8	56.4

S, susceptible; I, intermediate resistant; R, resistant.

### 3.2 Carbapenemase-encoding Elements Harbored by Clinical CRE Strains

The CRE strains were further tested for their ability to produce carbapenemase and carriage of carbapenemase genes. A total of 887 out of the 1105 CRE were found to produce carbapenemases. All these carbapenemase-producing CRE were found to carry different carbapenemase genes. The degree of correlation between carbapenem resistance phenotype and carriage of carbapenemase genes was over 90% for *K. pneumoniae* and *E. coli*, whereas only 80% of carbapenem-resistant *E. cloacae* strains were found to harbor carbapenemase genes. Among the CRE strains tested, the KPC-2-type carbapenemase gene (*bla*_KPC-2_) was the most dominant type and detected in 627 (57%) strains, whereas the *bla*_NDM_ gene was detected in 343 (31%) strains; 21 strains were found to harbor both genes (1.9%). The *bla*_IMP-4_ gene was detected in 35 (3%) strains, one of which was found to harbor the *bla*_KPC-2_ gene (0.1%) (Table 1). The prevalence of these three types of carbapenemase genes varied in different species of CRE. The *bla*_KPC-2_ gene was detectable in 517 of 703 (73%) *K. pneumoniae* isolates tested but only in 65 of the 164 (40%) *E. coli* isolates, and 19 of the 132 (14%) *E. cloacae* strains tested. The detection rate of *bla*_KPC-2_ was also < 30% in the species of *E. aerogenes*, *K. oxytoca*, *S. marcescens* and *C. freumdii*. On the other hand, the *bla*_NDM_ gene was detected in 67% of *E. aerogenes* (16/24) and *S. marcescens* (14/21), followed by *C. freumdii* (62%, 13/20), *E. cloacae* (61%, 81/132), *E. coli* (49%, 83/171), *K. oxytoca* (42%, 10/24) and *K. pneumoniae* (17%, 121/702). The *bla*_IMP-4_ gene was detected in several different species of CRE even though the prevalence rate was very low (Table 1).

### 3.3 Distribution of ST and PFGE Types Among Clinical Carbapenemase-producing CRE Isolates

**Fig. 1:**
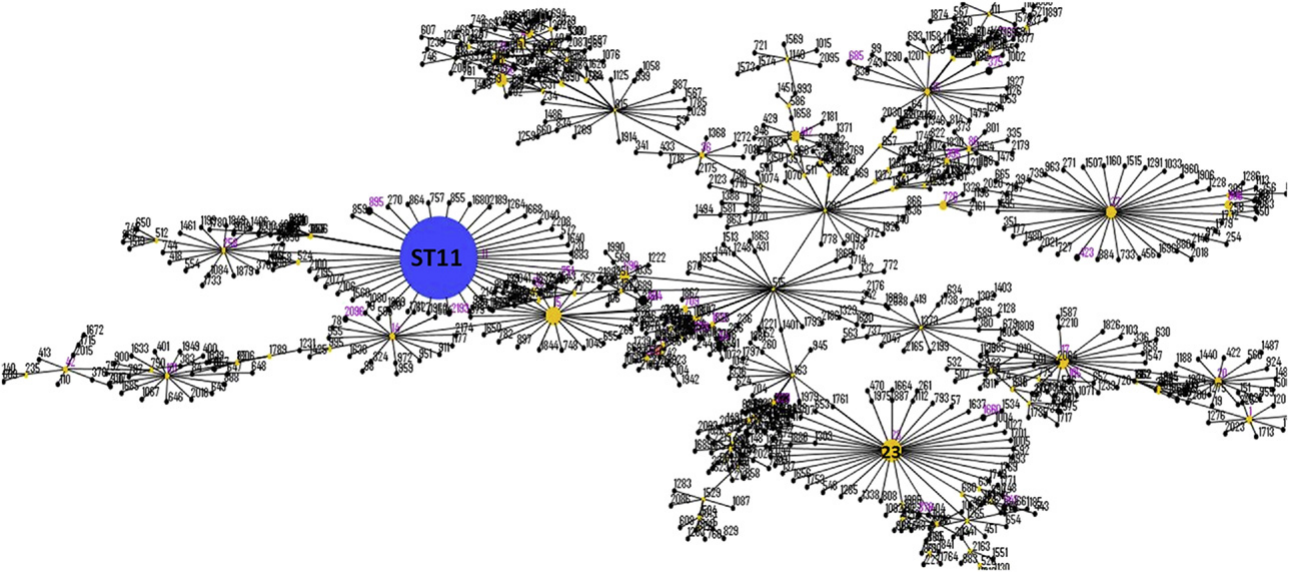
Minimal spanning tree based on multi-locus sequence typing of carbapenem-resistant *K. pneumoniae*. Colored circles and numbers represent different sequence types that have been detected in this study; the size of the circle is proportional to the numbers of the strains belonging to each type.

**Fig. 2:**
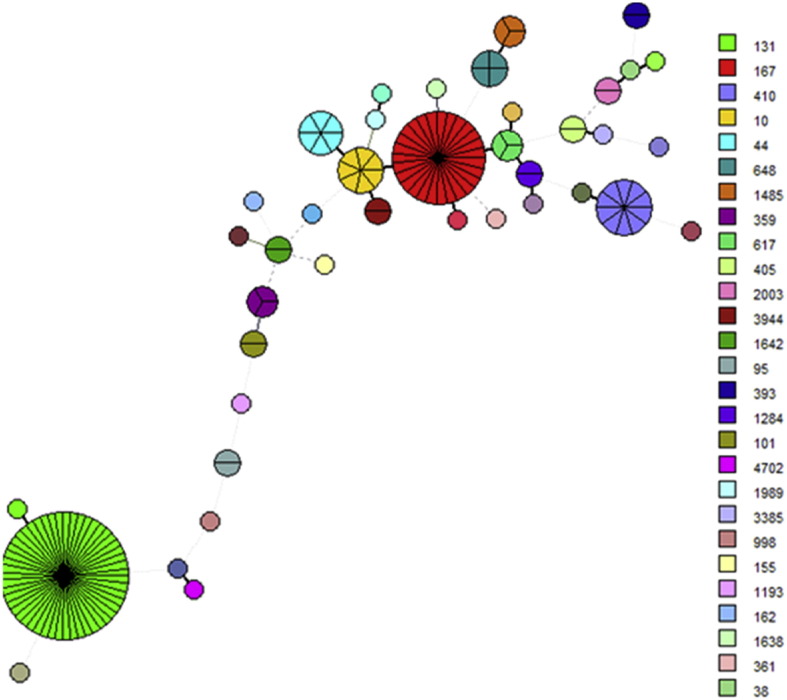
Minimal spanning tree based on multilocus sequence typing of carbapenem-resistant *E. coli*. Colored circles represent different sequence types; the size of the circle is proportional to the number of the strains belonging to each type.

Among the 668 carbapenemase-producing *K. pneumoniae* isolates that harbored carbapenemase genes, a total of 76 ST strain types were identified, with ST11 being the major type (Fig. 1, Table 3). The top 10 STs of *K. pneumoniae* were listed in Table 3. Unlike other parts of the world, where *K. pneumoniae* ST258 is the major type that produces carbapenemases, ST11 is the key strain type in China, accounting for as much as 60% of the carbapenemase-producing *K. pneumoniae* strains tested in this study. A total of 76 different PFGE patterns were observed among the 370 ST11 strains with 26 ST11 strains being untypable, suggesting that both clonal and non-clonal dissemination played an important role in the transmission of carbapenem resistant ST11 *K. pneumoniae* strains in China (**SF1**). Among the 150 clinical carbapenemase-producing *E. coli* isolates examined, 39 ST strain types were detected, with ST131, which accounted for 33% of the isolates, being the most dominant (Fig. 2, Table 3). It should be noted that ST167 and ST410 were the second and third most prevalent strain types, accounting for 17% and 7% of all *E. coli* isolates, respectively. A total of 89 PFGE patterns were observed among the 150 carbapenemase-producing *E. coli* isolates, suggesting that non-clonal dissemination played an important role in the transmission of carbapenemase-producing *E. coli* strains in China (**SF2**). PFGE patterns among the ST131 *E. coli* strains were less divergent compared to other strain types of *E. coli*. A total of 22 PFGE patterns were observed among the 52 ST131 strains, suggesting that both clonal and non-clonal dissemination contributed to carbapenemase-producing ST131 *E. coli* transmission in China (**SF2**). Another interesting observation is the close association between specific ST types and carriage of specific carbapenemase genes. Most of the ST types of *K. pneumoniae*, including ST11, were found to carry *bla*_KPC-2_, whereas ST23 (25/27) and ST17 (9/11) and ST45 (5/6) types of *K. pneumoniae* mainly carried *bla*_NDM_. On the other hand, ST35 and ST37 of *K. pneumoniae* were found to harbor both *bla*_NDM_ and *bla*_KPC-2_. A similar phenomenon was observed in the carbapenem-resistant *E. coli* isolates; for instance, ST131 (48/52), ST44 (5/6) and ST648 (3/4) types of *E. coli* mainly carried *bla*_KPC-2_, whereas other strain types such as ST167 (23/27), ST410 (10/10) and ST10 (7/7) mainly carried *bla*_NDM_ (Table 3).

**Table 3:** Top 10 STs of clinical carbapenemase-producing *K. pneumoniae* and *E. coli* strains and the carbapenemase genes they harbored.

*K. pneumoniae* (668)					*E. coli* (150)				
ST	No. of strains	%	*bla*_KPC-2_	*bla*_NDM-1_	ST	No. of strains	%	*bla*_KPC-2_	*bla*_NDM-1_
ST11	396	59.8	390	9	ST131	52	34.0	48	4
ST23	27	4.1	1	26	ST167	27	17.7	4	23
ST15	17	2.6	15	2	ST410	10	6.5	0	10
ST37	13	2.0	6	7	ST10	7	4.6	0	7
ST147	12	1.8	11	1	ST44	6	3.9	5	1
ST17	11	1.7	2	8	ST648	4	2.6	3	1
ST353	11	1.7	11	0	ST617	3	2.0	0	3
ST35	10	1.5	4	5	ST1485	3	2.0	0	3
ST268	8	1.2	1	0	ST359	3	2.0	1	2
ST420	7	1.1	7	0	ST95	2	1.3	0	1

Some strains may harbor more than one carbapenemase gene.

### 3.4 Prevalence and Characteristics of CRE in Different Parts of China

**Fig. 3:**
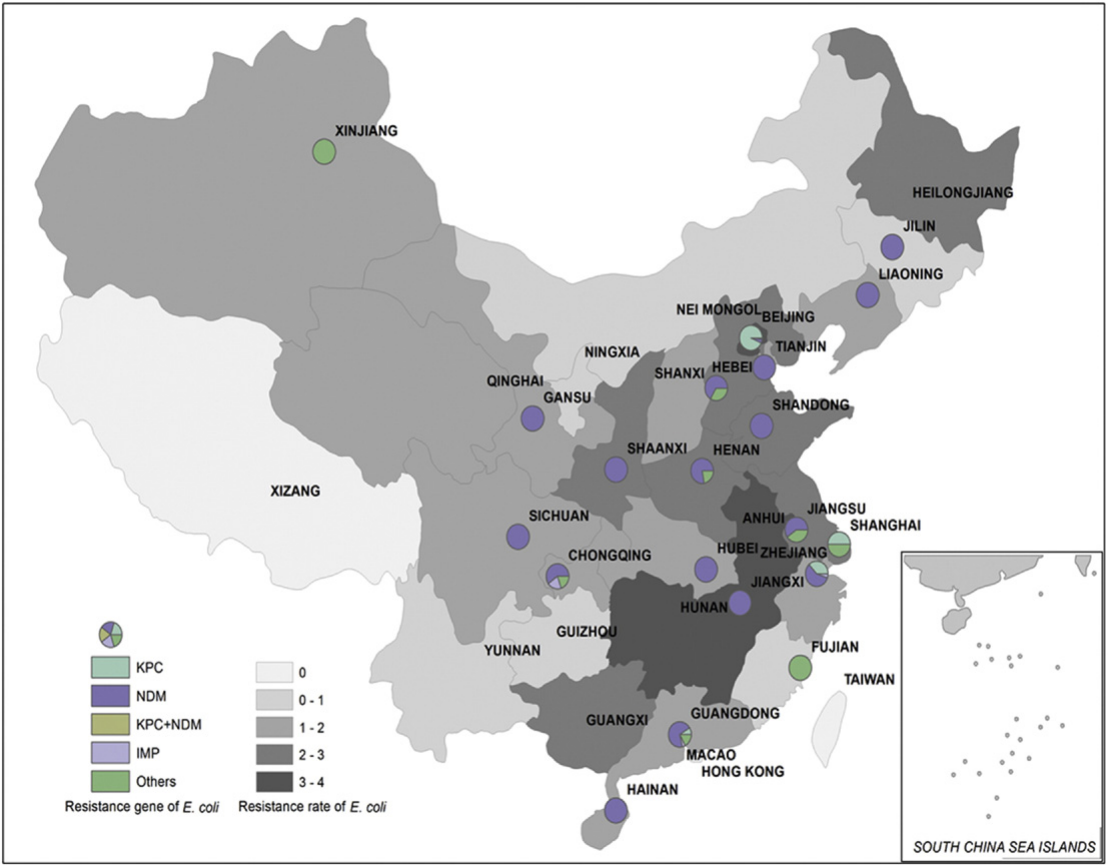
Carbapenem resistance rate and relative prevalence of various carbapenemase-producing elements among clinical carbapenem-resistant *E. coli* strains recovered from different provinces of China. Different background colors represent different rates of carbapenem resistance. Circle partitions represent the prevalence of different carbapenemases produced by carbapenem-resistant *E. coli* strains recovered from different locations. The resistance rate for each location was adopted from data of China Antimicrobial Resistance Surveillance Report (http://www.yiyimama.com/Sys/res/file/201512/20151220130152_4645_81ed026304834a5d81842924c78a1a9f_2014%E7%BB%86%E8%8F%8C%E8%80%90%E8%8D%AF%E7%9B%91%E6%B5%8B%E6%8A%A5%E5%91%8A.pdf; http://www.yiyimama.com/Sys/res/file/201512/20151220130134_7741_482f3b7ae95841998a37898e2ab2fa87_2015%E5%B9%B4%E7%9B%91%E6%B5%8B%E6%8A%A5%E5%91%8A.pdf).

**Fig. 4:**
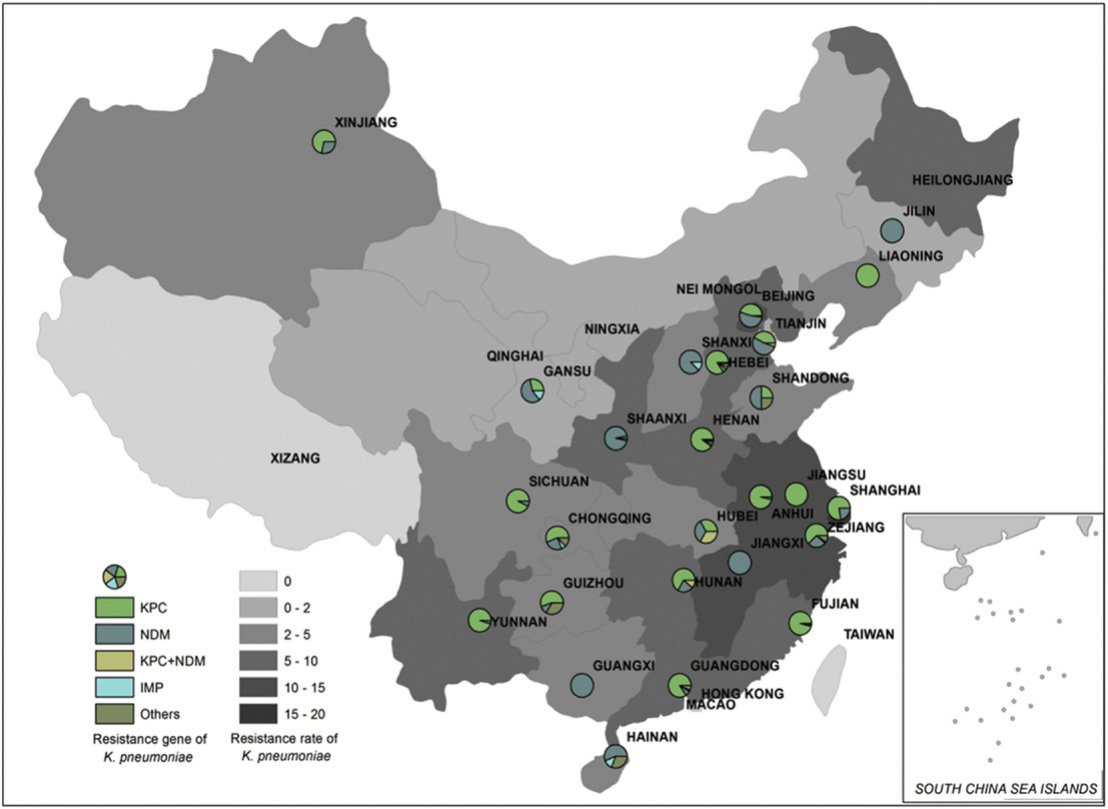
Carbapenem resistance rate and relative prevalence of various carbapenemase-producing elements among clinical carbapenem-resistant *K. pneumoniae* strains recovered from different provinces of China. Different background colors represent different prevalence levels of carbapenem resistance. Circle partitions represent the prevalence of different carbapenemases produced by carbapenem-resistant *K. pneumoniae* strains at different locations. The resistance rate for each location was adopted from data of China Antimicrobial Resistance Surveillance Report (http://www.yiyimama.com/Sys/res/file/201512/20151220130152_4645_81ed026304834a5d81842924c78a1a9f_2014%E7%BB%86%E8%8F%8C%E8%80%90%E8%8D%AF%E7%9B%91%E6%B5%8B%E6%8A%A5%E5%91%8A.pdf; http://www.yiyimama.com/Sys/res/file/201512/20151220130134_7741_482f3b7ae95841998a37898e2ab2fa87_2015%E5%B9%B4%E7%9B%91%E6%B5%8B%E6%8A%A5%E5%91%8A.pdf).

Data obtained from the China Antimicrobial Resistance Surveillance Report showed that the rate of carbapenem resistance in clinical *E. coli* and *K. pneumoniae* strains was around 0.6–3.6% and 1.2%–18.9% respectively in different provinces of China (http://www.yiyimama.com/Sys/res/file/201512/20151220130152_4645_81ed026304834a5d81842924c78a1a9f_2014%E7%BB%86%E8%8F%8C%E8%80%90%E8%8D%AF%E7%9B%91%E6%B5%8B%E6%8A%A5%E5%91%8A.pdf; http://www.yiyimama.com/Sys/res/file/201512/20151220130134_7741_482f3b7ae95841998a37898e2ab2fa87_2015%E5%B9%B4%E7%9B%91%E6%B5%8B%E6%8A%A5%E5%91%8A.pdf). In this study, variations in the prevalence rate of different carbapenemase genes were observed among different CREs recovered from different locations. For *K. pneumoniae* isolates, although the *bla*_KPC-2_ gene was prevalent among *K. pneumoniae* isolates in most parts of China, *bla*_NDM_ was the major resistance gene detectable in several regions such as Shanxi, Shaanxi, Guangxi, Jiangxi and Jilin. Second, both *bla*_KPC-2_ and *bla*_NDM_ were detectable in some parts of China such as Gansu, Beijing, Tianjin, Shanghai, Jiangshu and Zhejiang, even though *bla*_KPC-2_ remained more prevalent (Table 4, Fig. 3). Among carbapenem-resistant *E. coli* strains, NDM-type carbapenemase was the most common enzyme produced except in Beijing, Shanghai and Sichuan, where the majority of carbapenem-resistant *E. coli* isolates were found to produce the KPC-2 carbapenemase. In Hunan province, the majority of carbapenem-resistant *E. coli* produced *bla*_IMP-4_ (Table 4, Fig. 4).

**Table 4:** Resistance rate, number, major STs and carbapenemase genes among clinical carbapenemase-producing *K. pneumoniae* and *E. coli* strains collected at different locations in China.

Locations in China	*K. pneumoniae*				*E. coli*			
	Resist. rate (%)^a^	No. isolates	ST	Resist. genes	Resist rate (%)	No. isolates	ST	Resist. genes
Anhui	13.3	27	11	*bla*_KPC_	2.3	–	–	–
Beijing	18.7	52	11	*bla*_KPC_	3.1	59	131	*bla*_KPC_
Chongqing	2.9	14	11/35	*bla*_KPC_	–	–	–	–
Fujian	7.0	47	11	*bla*_KPC_	0.5	1	–	–
Gansu	1.1	7	17	*bla*_NDM_	1.0	9	410	*bla*_NDM_
Guangdong	4.0	54	11	*bla*_KPC_	1.4	21	10/167	*bla*_NDM_
Guangxi	2.5	1	11	*bla*_NDM_	1.2	–	–	–
Guizhou	4.6	6	29	*bla*_KPC_	0.8	–	–	–
Hainan	3.9	13	273	*bla*_NDM_	1.2	4	10/167	*bla*_NDM_
Hebei	8.7	52	11	*bla*_KPC_	2.3	3	410	*bla*_NDM_
Henan	15.3	89	11	*bla*_KPC_	2.7	7	167	*bla*_NDM_
Hubei	9.8	12	268	*bla*_IMP4_	1.2	2	410	*bla*_NDM_
Hunan	6.9	9	11	*bla*_KPC_	1.3	1	95	*bla*_IMP4_
Jilin	2.2	7	11	*bla*_NDM_	1.3	1	155	*bla*_NDM_
Jiangxi	13.5	7	11	*bla*_KPC_	3.0	1	–	–
Liaoning	7.6	27	11	*bla*_KPC_	1.5	3	167	*bla*_NDM_
Nanjing	12.7	8	11	*bla*_KPC_	2.2	3	167	*bla*_NDM_
Shandong	5.2	4	17	*bla*_NDM_	3.9	6	167	*bla*_NDM_
Shanxi	2.1	8	45	*bla*_NDM_	0.7	–	–	–
Shaanxi	8.8	32	23	*bla*_NDM_	5.7	7	131	*bla*_NDM_
Shanghai	20	45	11	*bla*_KPC_	2.5	2	405/648	*bla*_KPC_
Sichuan	3.4	14	147	*bla*_KPC_	2.0	4	167	*bla*_KPC_
Tianjing	1.9	19	35	*bla*_NDM_	1.0	5	167	*bla*_NDM_
Xinjiang	4.2	6	494	*bla*_KPC_	3.2	–	–	–
Yunnan	8.6	20	11	*bla*_KPC_	–	–	–	–
Zhejiang	12.3	89	11	*bla*_KPC_	1.8	12	167	*bla*_NDM_
Total		668				150		

a Resistance rate for each location was adopted from data of 2014 China Antimicrobial Resistance Surveillance Report (http://www.yiyimama.com/Sys/res/file/201512/20151220130152_4645_81ed026304834a5d81842924c78a1a9f_2014%E7%BB%86%E8%8F%8C%E8%80%90%E8%8D%AF%E7%9B%91%E6%B5%8B%E6%8A%A5%E5%91%8A.pdf; http://www.yiyimama.com/Sys/res/file/201512/20151220130134_7741_482f3b7ae95841998a37898e2ab2fa87_2015%E5%B9%B4%E7%9B%91%E6%B5%8B%E6%8A%A5%E5%91%8A.pdf).

For carbapenemase-producing *K. pneumonia*, the ST11 strain type was prevalent in most parts of China except the provinces of Gansu, Guizhou, Hainan, Shanxi, Shaanxi and Sichuan (Table 4). It should be noted that ST23 was the predominant type in Shaanxi, where 26 out of a total 32 *K. pneumoniae* isolates surveyed belonged to this strain type. All these ST23 strains were found to belong to the same clone as they exhibited identical PFGE pattern, again suggesting that clonal dissemination is common in certain regions. It is interesting to note that the low prevalence rate of ST11 type *K. pneumoniae* strains in these areas correlated with the low rate of recovery of the *bla*_KPC-2_ gene in such locations (Table 4). For carbapenemase-producing *E. coli*, ST131 was commonly detected in Beijing, with 44/59 *E. coli* isolates belonging to this strain type. The predominance of ST131 in Beijing correlated well with the high prevalence of KPC-2-producing *E. coli* isolates in this location, where 48 out of the 52 ST131 *E. coli* isolates tested were found to produce the KPC-2-type carbapenemase. Although ST131 is the most common clinical strain type, it is not widely distributed throughout the country. In comparison, ST167 and ST410 seem to be of greater concern since they are widely disseminated in China and known to cause infections nationwide (Table 4).

### 3.5 Mechanisms of Fosfomycin and Colistin Resistance in CREs

All fosfomycin-resistant *K. pneumoniae* and *E. coli* isolates were subjected to screening for the presence of the *fosA3* gene. Among the 410 fosfomycin-resistant *K. pneumoniae* isolates tested, 244 (59%) were found to harbor the *fosA3* gene; on the other hand, all the 17 fosfomycin-resistant *E. coli* isolates were positive for *fosA3*, suggesting that this gene plays a key role in mediating fosfomycin resistance in CREs in China. All CREs with colistin MIC ≧ 2 μg/ml were then subjected to screening for the presence of newly discovered colistin resistance gene *mcr-1*. Only two carbapenem-resistant *E. coli* (CREC) strains, CREC-A6 and CREC-TJ2, were found to harbor the *mcr-1* gene and exhibit colistin resistance. To determine if some colistin susceptible CRE strains might also carry *mcr-1*, we performed PCR screening of this gene for all the colistin susceptible CRE. Our data revealed that none of the colistin susceptible CRE strain carried the *mcr-1* gene, suggesting that the carriage of this gene was consistent with its colistin resistance phenotype. Please note that due to the large numbers of carbapenem-susceptible *Enterobacteriaceae*, we did not include these strains for the screening of *mcr-1*. Both CREC-A6 and CREC-TJ2 were subjected to further analysis by conjugation, S1-PFGE and Southern-hybridization. The plasmids harboring *mcr-1* in CREC-A6 and CREC-TJ2 were conjugative and found to be ~ 33 kb and ~ 60 kb in sizes respectively (Table 5). Illumina contigs were obtained for these two plasmids and subjected to BLASTN analysis with results showing that the ~ 33 kd conjugative plasmid recovered from CREC-A6 was highly similar to a *mcr-1*-bearing plasmid isolated from an *E. coli* strain recovered from farm animals in Estonia (NCBI accession no. KU743383), as well as other plasmids derived from *E. coli* of animal origin, pECJC-B65-33 (KX084392.1), while the ~ 60 kb, *mcr-1*-bearing plasmid from CREC-TJ2 was highly similar to the original *mcr-1*-bearing plasmid pHNSHP45 (KP347127.1) (Liu et al., 2016) (**SF3**).

**Table 5:** Characteristics of carbapenemase genes or *mcr-1*-bearing conjugative plasmids recovered from CREs.

CRE types	Carbapenemase/MCR-1	Total no.	No. of strains which harbored conjugative plasmids (rate)	Size (no.) of conjugative plasmids
*K. pneumoniae*	*bla*_NDM-1_	50	45 (90%)	35–54 kb (41), 140 kb (2), 450 kb (2)
	*bla*_KPC-2_	100	25 (25%)	60–70 kb (6), 85–95 kb (6), 120–130 kb (7), 210–230 kb (6)
*E. coli*	*bla*_NDM-1_	50	47 (94%)	35–54 kb (44), 110 kb (3)
	*bla*_KPC-2_	50	45 (90%)	60–70 kb (15), 75–85 kb (5), 85–95 kb (6), 120–130 kb (9), 210–230 kb (10)
	*mcr-1*	2	2 (100%)	33 kb (1), 60 kb (1)

### 3.6 Mechanisms of Transmission of Carbapenemase Genes Among Clinical CRE Strains

To understand the mechanisms underlying the transmission of carbapenemase genes among CREs, conjugation experiments were performed on 100 *K. pneumoniae* strains carryin*g* the *bla*_KPC-2_ element, 50 *K. pneumoniae* strains carrying *bla*_NDM_, and each of 50 strains of *E. coli* carrying *bla*_KPC-2_ and *bla*_NDM_ respectively. Conjugation rate was very high among *bla*_NDM_-bearing plasmids in both carbapenem-resistant *K. pneumoniae* (CRKP) and *E. coli*. On the other hand, the conjugation rate of the *bla*_KPC-2_-bearing plasmids in *E. coli* was high, but relatively low in *K. pneumoniae* (Table 5).

**Fig. 5:**
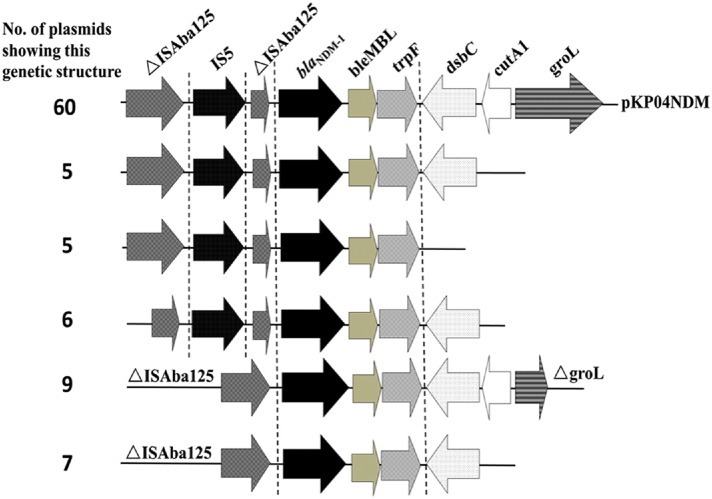
Number of plasmids carrying diverse structures of *bla*_NDM-1_-bearing mobile elements in conjugative plasmids harbored by carbapenemase-producing clinical *K. pneumoniae* and *E. coli* strains.

For *bla*_NDM_-bearing plasmids in both *K. pneumoniae* and *E. coli*, the majority of plasmids (85/92 or 92%) were at sizes of 35 kb–60 kb and shown to belong to IncX3 through plasmid typing. Other *bla*_NDM_ -bearing plasmids with sizes of ~ 110 kb were detected in *E. coli*, whereas similar plasmids of ~ 140 kb and ~ 450 kb could be recovered from *K. pneumoniae* (Table 5). PCR mapping of the conservative regions of IncX3 plasmid and regions carrying *bla*_NDM_-bearing mobile elements showed that all IncX3 conjugative plasmids recovered from these CRE strains contained a similar IncX3 backbone, but slightly different mobile elements. A total of six different *bla*_NDM_ genetic environments were found and shown in Fig. 5. Genetic variations were detectable in the upstream transposase genes and several downstream genes. This core structure was also detectable in other non-IncX3 conjugative plasmids carrying the *bla*_NDM_ gene (Fig. 5).

**Fig. 6:**
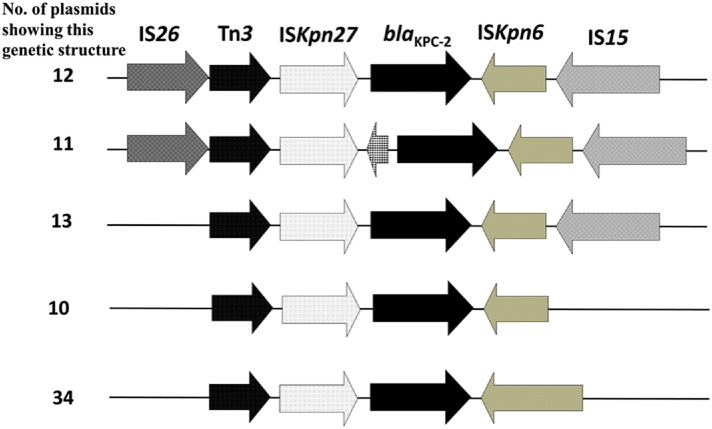
Number of plasmids carrying diverse structures of *bla*_KPC-2_-bearing mobile elements in conjugative plasmids harbored by carbapenemase-producing clinical *K. pneumoniae* and *E. coli* strains.

Most of the *bla*_KPC-2_-bearing plasmids (90%) in *E. coli* were transmissible, but the conjugative rate of this type of plasmids was relatively low among *K. pneumoniae* strains (25%). S1-PFGE and Southern hybridization revealed that conjugative plasmids harboring *bla*_KPC-2_ were genetically more divergent than those carrying *bla*_NDM_, and can be categorized into following groups, ~ 60 to ~ 70 kb, ~ 85 to ~ 95 kb, ~ 100 to ~ 110 kb, ~ 120 to ~ 130 kb and ~ 210 to ~ 230 kb (Table 5). The genetic environment of *bla*_KPC-2_ in these conjugative plasmids, analyzed by primer walking was found to share a similar core structure, IS*Kpn27*-*bla*_KPC-2_-IS*Kpn2*, implying that this mobile element played a key role in the transmission of *bla*_KPC-2_ gene (Fig. 6).

## 4 Discussion

Findings of this work confirmed that production of carbapenemases is the major mechanism of carbapenem resistance in CRE in China, among which NDM and KPC-2 were the major carbapenemases concerned. These data provide important insight into the development anti-CRE therapy in China, and demonstrated that the types of resistance genes harbored by CRE strains differed significantly from one region to another. In particular, our data contradicted those of a recent meta surveillance conducted in European countries, which showed that only 71% of CRE were carbapenemase-producing, among which a wide variety of carbapenemases were detected (Grundmann et al., 2016).

Although there is no evidence to suggest that CRE originated from China, dissemination of such strains in China was found to be extremely rapid, eventually facilitating transmission to other parts of the world through traffic and trade. This study represents the first comprehensive nationwide surveillance of CRE in China to provide an overview of the genetic and phenotypic features of this category of multidrug resistant organisms in the country. The data is highly representative as the surveillance covers almost every province or municipal city. Key findings of this study are summarized as follows. First, *bla*_KPC-2_ and *bla*_NDM_ were found to be the key carbapenemase genes responsible for mediating development of the carbapenem resistance phenotypes in CREs in China, with *bla*_KPC-2_ being the most common carbapenemase gene harbored by *K. pneumoniae*; however, *bla*_NDM_ was found to be the predominant resistance gene in *E. coli*. Other carbapenemase genes such as *bla*_IMP-4_ were much less frequently detected; other elements such as *bla*_VIM_ and *bla*_OXA-48_ were not detectable in a single clinical CRE strain tested in this work. Second, 21 out of 1105 CRE strains tested were found to carry multiple carbapenemase genes, in particular the *bla*_NDM_ and *bla*_KPC-2_ combination, with *K. pneumoniae* being the most common species in this category. These observations urged the development of novel therapies to combat CRE in China. Current new antibiotics such as ceftazidime-avibactam may not be suitable to treat CRE in China since a large proportion of CRE produce NDM carbapenemase, to which ceftazidime-avibactam is not active. Development of inhibitors targeting NDM and KPC-2 should be a feasible strategy to develop novel anti-CRE therapies.

Although CRE exhibiting a range of strain types were detected in China, several unique strain types are clearly responsible for the increased rate of CRE infections in China. ST11 of *K. pneumoniae*, but not ST258, a common strain type reported worldwide, is the major strain type, which exhibited signs of multi-clonal dissemination. Other strain types were relatively rare and more sporadic, with ST23 (4%) and ST17 (2.6%) being the 2nd and 3rd most common strain types. Except for some regions, ST11 is the major CRKP in China. Genetic analysis showed that ST258 is not simply a distinct clone of ST11. These two types of CRKP shared about 3/4 of the common genome, while 1/4 of the ST258 genome is derived from other types of *K. pneumonia* (Liang Chen et al., 2014). The genetic basis of predominant prevalence of ST11 in China and ST258 in other part of the world is not well understood. Unlike ST11, which was strictly associated with KPC-2 production, other strain types including ST23, ST17, ST76 and ST45 were associated with NDM production. Further research is needed to understand the adaptability of plasmids encoding different carbapnemases in different strain types of *K. pneumoniae*. Similar to the case of *K. pneumoniae* among which ST11 was the major strain type, a major strain type of carbapenemase-producing *E. coli* was also identified. ST131 of CREC was found to be a major strain type in both China and other countries. Consistent with reports in other parts of the world (Cai et al., 2014, Naas et al., 2011, Ortega et al., 2016), ST131 in this study was also closely associated with KPC-2 production. However, it should be noted that ST131 type *E. coli* were more commonly detected in Beijing and Zhejiang province where signs of clonal spread were evident, whereas it was less common in other parts of the country. In contrast, ST167 and ST410 seem to be of more clinically relevant since they are not only widely disseminated in China but are also the major *bla*_NDM_-bearing strains. Further dissemination of these types of carbapenem-resistant *E. coli* may become a significant problem in clinical settings in China. Variation between the prevalence rate of CRE among different locations was obvious. The unique pattern of distribution CRE and carbapenemase genes may suggest that clonal spread in specific region is common.

Consistent with the sporadic reports in China, characterization of the *bla*_KPC-2_- and *bla*_NDM_-bearing plasmids recovered from CRE in this study indicated that IncX3 conjugative plasmids carrying *bla*_NDM_ is the major gene involved in dissemination of *bla*_NDM_ among clinical CRE strains (Yang et al., 2014, Yang et al., 2015, Zhang et al., 2016). Although other conjugative plasmids were also involved in the transmission of *bla*_NDM_, the core structure of such mobile elements remains highly similar, suggesting that horizontal transfer of such mobile elements is the major mechanism responsible for emergence and rapid transmission of *bla*_NDM_. Compared to *bla*_NDM_, *bla*_KPC-2_-bearing plasmids were structurally more divergent, as *bla*_KPC-2_-bearing plasmids of various types and sizes were detectable in both *K. pneumoniae* and *E. coli*. However, the major mobile element that harbored *bla*_KPC-2_ was also found to be highly conservative, suggesting that this mobile element plays an important role in the emergence and transmission of *bla*_KPC-2_ among clinical CRE strains. The fact that the *bla*_KPC-2_-bearing plasmids in *K. pneumoniae* were less conjugative (35% were conjugative), but such plasmids in *E. coli* could undergo conjugation in most cases, suggests that these plasmids most likely emerged in *K. pneumoniae*, and were transmitted to *E. coli* and other bacterial species through conjugation.

In conclusion, this study reported the first nationwide surveillance of CRE in China, a largest scale of CRE surveillance that have ever been reported. Essential information obtained from this study include: 1) *bla*_NDM_ and *bla*_KPC-2_ were the major carbapenemase genes harbored by clinical CRE strains, with *bla*_KPC-2_ being more prevalent in *K. pneumoniae* and *bla*_NDM_ being more prevalent in *E. coli*; 2) ST11 was the dominant type of CRKP, while ST131, ST167 and ST410 were the dominant types of CREC; 3) Polymyxins remained effective for *K. pneumoniae* and *E. cloacae*, while a number of antibiotics are still effective for treatment of *E. coli* infections; 4) IncX3 plasmid was the major type of plasmid mediating transmission of *bla*_NDM_ among clinical CRE strains, whereas plasmids harboring *bla*_KPC-2_ were more diverse in structure; 5) regardless of the structural diversity of plasmids harboring carbapenemase genes, the core structures of mobile elements containing *bla*_NDM_ and *bla*_KPC-2_ were highly conservative; 6) the horizontal transfer of core structure of mobile elements carrying *bla*_NDM_ and *bla*_KPC-2_ responsible for the transmission of these two carbapenemase genes in clinical CRE in China.

## Financial Support

This study was funded by grants provided by the National Basic Research (973) Program of China (2013CB127200) and Collaborative Research Fund from Research Grant Council (C7038-15G and C5026-16G).

## Conflicts of Interest

All authors: No reported conflicts.

## Author Contribution

RZ designed research and collected all the CRE strains; LZL performed molecular characterization of all CRE strains; HWZ, JPL, YF, YL and KL performed CRE strain identification and phenotypic characterization; EWCC analyzed the data and contributed to manuscript writing; SC designed the research, supervised the progress of the study and wrote the manuscript.
